# The antimicrobial activity of prototype modified honeys that generate reactive oxygen species (ROS) hydrogen peroxide

**DOI:** 10.1186/s13104-014-0960-4

**Published:** 2015-01-28

**Authors:** Jonathan Cooke, Matthew Dryden, Thomas Patton, James Brennan, John Barrett

**Affiliations:** Centre for Infection Prevention and Management, Division of Medicine, Imperial College, Hammersmith Campus, London, W12 0NN UK; Manchester Pharmacy School, Faculty of Medical and Human Sciences University of Manchester, Manchester, UK; Hampshire Hospitals NHS Foundation Trust, Department of Microbiology, Romsey Road, Winchester, SO22 5DG UK; Rare and imported pathogens laboratory (RIPL), Public Health England, Manor Farm Road, Porton Down, Wiltshire, SP4 0JG UK; Institute of Technology, Ash Lane, Sligo, Ireland

**Keywords:** Reactive oxygen species (ROS), Honey, Modified honey, Hydrogen peroxide, Antimicrobial, Wound dressings

## Abstract

**Background:**

Antimicrobial resistance continues to be a global issue in healthcare organisations. Honey has long been shown to possess wound healing and antimicrobial properties that are dependent on a number of physical and chemical properties of the honey. We tested the antimicrobial activity of a medicinal honey, *Surgihoney®* (SH) and two prototype modified honeys made by *Apis mellifera* (honeybee) against *Staphylococcus aureus* (NCIMB 9518). We also examined the modified honey prototypes for the ability to generate reactive oxygen species (ROS) by changing the level of production of hydrogen peroxide from the samples.

**Methods:**

*Surgihoney®* (SH) was compared with two modified honeys, Prototype 1 (PT1) and Prototype 2 (PT2) using a bioassay method against a standard strain of *Staphylococcus aureus.* Further work studied the rate of generation of ROS hydrogen peroxide from these preparations.

**Results:**

*Surgihoney®* antimicrobial activity was shown to be largely due to ROS hydrogen peroxide production. By modification of *Surgihoney®,* two more potent honey prototypes were shown to generate between a two- and three-fold greater antibacterial activity and up to ten times greater ROS peroxide activity.

**Conclusions:**

*Surgihoney®* is a clinically available wound antiseptic dressing that shows good antimicrobial activity. Two further honey prototypes have been shown to have antimicrobial activity that is possible to be enhanced due to demonstrated increases in ROS peroxide activity.

## Background

Resistance to antimicrobials (antibacterials, antifungals and antivirals) is now a global concern [1,2], leading to governments developing antimicrobial resistance (AMR) strategies that include programmes of antimicrobial stewardship [1]. AMR has considerable clinical and financial consequences and the financial burden of AMR is likely to be considerably underestimated [3]. There is is now universally discouragement of the use of agents that are used systemically being applied topically to skin infections. The development of effective non-toxic topical antimicrobial agents is being actively pursued [4].

Honey has been used as a topical antiseptic for at least 5,000 years [5,6]. The antimicrobial activity of honeys is thought to be due to the physical nature, pH and hyperosmolarity of the preparations and antimicrobial components of some honeys which include methylglyoxal, bee defensin-1 and hydrogen peroxide [7-11]. Furthermore, there have been no reports of any loss of antimicrobial activity of honeys due to the development of antimicrobial resistance [12].

We report here in-vitro results of testing a new biologically modified honey, *Surgihoney®*, that has been shown to have antimicrobial activity in-vitro and clinical effectiveness in the treatment of acute and chronic wounds and the prevention of surgical wound infections [13,14]. *Surgihoney®* is sourced from any honey that meets the standard of zero tolerance for both the presence of antibiotics and pesticides. The European standard for the former, is less than 10 ppb of antibiotic residues that then meets a criteria of “no antibiotics detected”. As a pre-requirement that not even low doses of antibiotics are introduced to wound tissue *Surgihoney®* requires a level of 0 ppb of both antibiotics and pesticide residues. Within this requirement the honey used to form Surgihoney is not floral source dependent. The honey source must confirm with the Codex Alimentarius definition of honey defined as “Honey consists essentially of different sugars, predominantly fructose and glucose as well as other substances such as organic acids, enzymes and solid particles derived from honey collection” [15]. The moisture content of honey should not be more than 20% for all honeys with the exception of heather honey than should have a moisture content no more than 23%. All nectars collected by the honey bee are a weak solution of sugars (largely sucrose) in water but contain generally large quantities of naturally occurring yeast which is then stored until dried to the point where there is no free water present in warm conditions in the bee hive. The requirement by the honey bee to be able to store carbohydrate for the winter months, which has not turned to alcohol has resulted in the nectar, once collected by the honey bee, showing the ability to resist fermentation, principally by the formation of reactive oxygen. The engineering process that has been developed to produce Surgihoney mimics this natural process in a highly controlled way with the formation of low levels of reactive oxygen in a stable way over an extended period of time

## Methods

### 1. Determination of honey activity by bioassay method

The antibacterial activity of *Surgihoney®* (SH) and two modified honeys, Prototype 1 (PT1) and Prototype 2 (PT2) was measured using *Staphylococcus aureus* (NCIMB 9518) and expressed as the equivalent % phenol. Values were calculated from the 9 determinations made for each sample, 3 samples per day on 3 separate days

#### Assay method

The agar well diffusion method used was adapted from the punch plate assay for inhibitory substances described in the Microbiology Standard Methods Manual for the New Zealand Dairy Industry (1982) [16].

#### Inoculum preparation

Overnight culture was adjusted to an absorbance of 0.5 measured at 540 nm using sterile nutrient broth as a blank and a diluent and a cuvette with a 1 cm pathway.

#### Assay plate preparation

A volume of 100 μl of the culture adjusted to 0.5 absorbance was used to seed 150 ml nutrient agar to make the assay plates. The agar was swirled to mix thoroughly and poured into large petri dishes which had been placed on a level surface. As soon as the agar was set the plates were placed upside down overnight before using the next day. For assay these seeded plates were removed from 4°C and allowed to stand at room temperature for 15 min before cutting 7.0 mm diameter wells into the surface of the agar. 250 μl of test material (sample or standard) was placed into each well.

#### Catalase solution

A 200 mg/ml solution of catalase from bovine liver (Sigma C9322, 2900 units/mg) in distilled water was prepared fresh each day.

#### Honey preparation

Primary sample solutions were prepared by adding 4 g of sample to 4 ml of distilled water and placed at 37°C for 30 min to aid mixing. To prepare secondary solutions, 2 ml of the primary sample solution was added to 2 ml of distilled water in universals and mixed for total activity testing and 2 ml of the primary sample solution was added to 2 ml of catalase solution and mixed for non-peroxide activity.

#### Preparation of phenol standards

Standards (w/v) 10%, 30%, 50% phenol were prepared by dissolving phenol in water. Phenol standards were brought to room temperature in the dark before use and were mixed thoroughly before addition to test wells. Each standard was placed in three wells to test in triplicate. Standards were kept at 4°C with an expiry date of one month.

#### Sample and standard application

All samples and standards were tested in triplicate by adding 250 μl to each of 3 wells.

#### Plate incubation

After application of samples the plates were incubated for 18 +/− 0.5 hours at 37°C. The diameter of inhibition zones, including the diameter of the well (7.0 mm), was recorded.

#### Calculation of antibacterial activity of samples

The mean diameter of the clear zone around each phenol standard was calculated and squared. A standard graph was plotted of % phenol against the square of the mean diameter of the clear zone. A best-fit straight line was obtained using linear regression and the equation of this line was used to calculate the activity of each diluted honey sample from the square of the mean measurement of the diameter of the clear zone. To allow for the dilution (assuming the density of the *Surgihoney®* to be 1.35 g/ml) this figure was multiplied by a factor of 4.69 and the activity of the samples was then expressed as the equivalent phenol concentration (% w/v).

### 2. Determination of honey activity by H_2_O_2_ method

The activity was measured using the *Merckoquant® 1.10011. & 1.10081.*

#### Peroxide test kits

Concentrations expressed as the equivalent mg/L H_2_O_2_.Samples were diluted 1:10 with purified water. Following 5 min incubation, all samples were measured for H_2_O_2_ production each hour over a 12 hour period followed by 24 and 48 hour time points.

#### Method of determination

Peroxidase transfers oxygen from the peroxide to an organic redox indicator, which is then converted to a blue coloured oxidation product. The peroxide concentration is measured semi-quantitatively by visual comparison of the reaction zone of the test strip with the fields of a colour scale. A hydrogen peroxide test strip is immersed into the Surgihoney® sample for a period of 1 second, allowing excess liquid to run off the strip onto an absorbent paper towel and after 15 seconds (For Catalogue No. 110011) and 5 seconds (For Catalogue No. 110011), after which the colour formed is compared with the manufacturer’s colour chart and the concentration of hydrogen peroxide in the honey is obtained’

## Results

### 1. Activity rating

The antimicrobial activity produced by the modification of the honey samples resulted in a two-fold and almost three-fold respectively increase in phenol activity with PT1 and PT2 compared with *Surgihoney®* alone. The results for the three samples of *Surgihoney®* (SH) and two modified prototypes, PT1 and PT2 are shown in Table 1. The non-peroxide activity is due to the osmotic and acidic effect of the preparation.

**Table 1:** **Showing the peroxide and non-peroxide antibacterial activities of** ***Surgihoney®*** **(SH) and two modified prototypes, PT1 and PT2 against** ***Staphylococcus aureus*** **(NCIMB 9518)**

**Sample name**	**Batch no.**	**Total activity**	**Non-peroxide activity**
		**(% phenol)** $\overline{\mathbf{X}}\left[\pm \boldsymbol{\upsigma} \right]$	**(% phenol)** $\overline{\mathbf{X}}\left[\pm \boldsymbol{\upsigma} \right]$
**Surgihoney**	**2015-06-018B**	**31.5 (2.4)**	**0.0 (0.0)**
**Surgihoney PT 1**	**HHI14110311**	**64.6 (3.0)**	**6.9 (1.0)**
	**HHI14110312**	**82.7 (3.5)**	**9.8 (2.2)**

### 2. Determination of honey activity by H_2_O_2_ method

**Figure 1:**
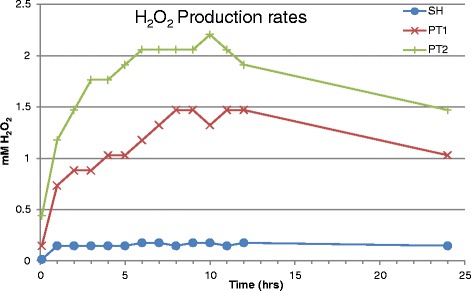
**Different hydrogen peroxide production rates for** ***Surgihoney®*** **(SH) and two modified prototypes, PT1 and PT2.**

**Figure 2:**
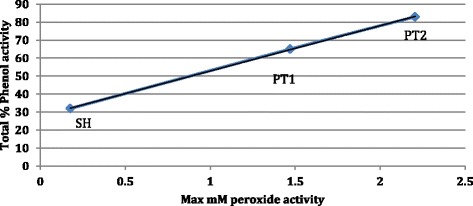
**Relationship between phenol activity and maximum hydrogen peroxide activity in modified honeys, SH, PT1 and PT2.**

The prototype modifications are observed to generate up to seven and ten times the ROS hydrogen peroxide activity of *Surgihoney®*. The results for the three samples are shown in Figure 1. By taking the maximum level of hydrogen peroxide output for each of the three honey prototypes and plotting this against the total phenol activity a linear relationship is observed (Figure 2).

## Discussion

*Surgihoney®* (SH), and its two prototypes, have been shown previously to possess potent in-vitro antimicrobial activity against a wide range of pathogenic and resistant Gram + ve and Gram –ve bacteria using a variety of techniques that included MIC and MBC determinations and time kill curves [17]. The results from the present work show that the main antimicrobial activity of *Surgihoney®* (SH) and two modified prototypes, PT1 and PT2 are due to ROS hydrogen peroxide. This is a similar finding to certain, but not all, honeys from a variety of floral sources [18-20].

However, unlike previous work the availability of ROS hydrogen peroxide from the samples is able to be enhanced and at 12 hours is seven and ten times respectively the value for *Surgihoney®* (SH) alone. There is a striking linear relationship between the antimicrobial activity and the maximum output of ROS hydrogen peroxide from the three honey prototypes.

This ROS peroxide activity offers potent antimicrobial activity that is ideally suited for a wound dressing that is applied to acute or chronic wounds to treat or prevent wound infections [21]. Whilst a small amount of catalase is present in wounds and serum level of catalase in males has been reported as 50 kU/l [22] it has been shown that catalase activity in healing wounds actually decrease during the first week post-wounding and activity levels of catalase recover to its original level at two weeks post-wounding [23]. Such concentrations of catalase are thus extremely unlikely to influence the antimicrobial activity observed with exogenously applied *Surgihoney®* or the two modified prototypes, PT1 and PT2.

Limitations of this study include the suitability of the method for determination of antibacterial activity. Thus a microdilution method might have been better than the agar well diffusion method. In other studies we have done both agar diffusion and microdilution MIC’s in other studies [13,17]. However these studies did not look at the ROS activity as was undertaken in the present study. Also it might have been helpful to add a control of another medical grade honey to be able to compare the activity of Surgihoney to other honey products that are available. Again such comparisons have been described previously [13,17].

The ideal characteristics for an antimicrobial wound dressing are: effectiveness, lack of toxicity, ease of use, patient and clinician acceptability and value for money [4].

ROS hydrogen peroxide is an effective antimicrobial and is already used as a biocide for its potent activity against vegetative bacteria [24], yeasts [25] and spores [26]. It produces its antimicrobial effect through chemical oxidation of cellular components [27].

The human toxicity of ROS hydrogen peroxide is concentration dependent and one study has claimed that the differential concentrations for antimicrobial and human toxicity might overlap [28]. By contrast, certain preparations of honey have been shown to be an effective antimicrobial agent by supplying low concentrations of ROS hydrogen peroxide to wounds continuously over time rather than as a large amount at the time of dressing and without such toxicity [29]. Indeed there is compelling evidence that where physiological levels of ROS hydrogen peroxide are applied to mammalian cells there is a stimulation of biological responses and activation of specific biochemical pathways in these cells [30].

*Surgihoney®* and the two modified prototypes, PT1 and PT2 are antimicrobial dressings that appear to offer effective ROS hydrogen peroxide release over at least 24 hours. More studies are needed to determine the kinetics and time kill for these preparations in order to predict the most effective period of dressing change to ensure optimal clinical and antimicrobial effects.

## Conclusions

*Surgihoney®* and the two modified prototypes, PT1 and PT2 have been shown to have potent antimicrobial activity against a standard strain of *Staphylococcus aureus.* These antimicrobial activities have been shown to be due to ROS hydrogen peroxide. The activity is scalable and can be described in terms of ROS hydrogen peroxide activity. These modified honeys offer a dressing that is effective, non-toxic and easy to administer.
